# Data for vancomycin elution, activity and impact on mechanical properties when incorporated into orthopedic bone cement

**DOI:** 10.1016/j.dib.2018.07.028

**Published:** 2018-07-17

**Authors:** Aaron R. Bishop, Sunjung Kim, Matthew Squire, Warren E. Rose, Heidi-Lynn Ploeg

**Affiliations:** a University of Wisconsin – Madison, Biomedical Engineering, United States; b University of Wisconsin – Madison, Mechanical Engineering, United States; c University of Wisconsin – Madison, School of Pharmacy, United States; d University of Wisconsin School of Medicine and Public Health, United States

## Abstract

In this article, we report data on the antibiotic elution and efficacy, and mechanical properties of Palacos bone cement with different amounts of added vancomycin (0.0, 0.125, 0.25, 0.5, 1.0, 2.0 g), see “*Vancomycin elution, activity and impact on mechanical properties when added to orthopedic bone cement”* (Bishop et al., 2018) [1]. Mechanical testing was performed for four-point bending, compression, and fracture toughness. The release characteristics of vancomycin was recorded for up to 60 days to estimate the elution profile. The eluted vancomycin efficacy at eliminating the four most common causative orthopedic implant pathogens is also reported.

**Specifications Table**

**Table:** 

Subject area	*Biomechanics, Pharmacy*
More specific subject area	*Orthopedic, Antimicrobial agent*
Type of data	*Image (X-ray, microscopy, etc.), figure, tabulated*
How data was acquired	*SEM (Zeiss-LEO,Oberkochen, Germany), MTS (Criterion C43.104, MTS Systems, Eden Prairie, MN), High performance Liquid Chromatography (HPLC)*
Data format	*Analyzed data*
Experimental factors	*Palacos bone cement different amounts of added vancomycin: 0.0, 0.125, 0.25, 0.5, 1.0, 2.0 g*
Experimental features	*Mechanical testing using MTS machine measured flexural modulus flexural strength, compressive modulus, compressive yield strength, and fracture toughness, according to ISO 5833. The drug elution test was determined using high performance liquid chromatography (HPLC) with a*$C_{18}$*column. Three cylindrical samples (6 mm diameter × 4.5 mm height) were sterilized by ethylene oxide gas and then submerged in 3.4 mL of tryptic soy broth inoculated with bacteria for each test condition for antimicrobial activity testing. Drug elution cements were stored in − 20* °*C freezer and all mechanical testing cements were wet cured in a phosphate-buffer solution (PBS) for 21 days at room temperature (22* °*C) before testing.*
Data source location	*Department of Mechanical Engineering and School of Pharmacy, University of Wisconsin Madison*
Data accessibility	*Data is with this article.*
Related research article	Bishop A.R., Kim S., Squire M.W., Rose W.E., Ploeg H., *Vancomycin elution, activity and impact on mechanical properties when added to orthopedic bone cement*, Journal of Mechanical Behavior of Biomedical Materials S1751–6161(18)30459-4, https://doi.org/10.1016/j.jmbbm.2018.06.033[1]

**Value of the data**

• These data are of value in cemented joint arthroplasty using Palacos with added vancomycin as a prophylactic measure against infection.
• The mechanical test data of wet cured samples demonstrated that mechanical properties of Palacos bone cement with upto 0.5 g of vancomycin met all ISO minimum requirements.
• The release characteristic test data showed that the elution profile is suited for clinical use since the maximum elution occurs during the critical first week after surgery and would effectively eliminate *S. aureus* contamination that may inadvertently occur during the surgical procedure.
• The antimicrobial activity test data showed that the eluted concentration from samples with greater than 0.25 g vancomycin per Palacos packet was sufficient to eliminate a 10^3^ colony forming unit per mL (CFU/mL) initial inoculum of *S. aureus*, including methicillin-resistant *S. aureus* (MRSA).

## 1 Data

The data provided here are

• Mechanical test data: flexural modulus, flexural strength, compressive modulus, compressive yield strength, and fracture toughness calculated from the force-displacement curves.
• Scanning electron microscope (SEM) images from the fracture surfaces of four-point bending samples.
• Release characteristic test data for vancomycin added to Palacos bone cement.
• Antimicrobial activity test data for eluted vancomycin efficacy at eliminating four most common causative orthopedic implant pathogens (MRSA n315, ATTC MRSA 33591, ATCC *S.aureus* 29213, and ATCC *S. epidermidis* 35984).

### 1.1 Mechanical testing data

See Table 1, Table 2, Table 3.

**Table 1:** Results from four-point bend testing. Results are reported as median ± 1 standard deviation.

**4 - Point bending test**							
**Cement**	**Antibiotic**	**Amount of antibiotic added [g]**	**Mixing**	**Conditioning**	**Testing condition**	**Bending modulus [MPa]**	**Bending strength [MPa]**
Palacos	–	–	Vacuum	Ambient, saline, 21 days	Ambient	2192 ± 164.2^1^	55.40 ± 3.531^3^
Palacos	Vancomycin	0.125	Vacuum	Ambient, saline, 21 days	Ambient	2349 ± 163.9^1^	57.72 ± 1.515^3^
Palacos	Vancomycin	0.25	Vacuum	Ambient, saline, 21 days	Ambient	2357 ± 301.0^1^	52.63 ± 2.221^3^
Palacos	Vancomycin	0.50	Vacuum	Ambient, saline, 21 days	Ambient	2267 ± 200.4^1^	56.71 ± 2.331^3^
Palacos	Vancomycin	1.0	Vacuum	Ambient, saline, 21 days	Ambient	2369 ± 64.00^1^	55.80 ± 1.541^3^
Palacos	Vancomycin	2.0	Vacuum	Ambient, saline, 21 days	Ambient	2038 ± 164.2^1^^,^^2^	46.80 ± 1.700^4^

1 Significantly higher than the ISO minimum requirement of 1800 MPa.

2 Significantly lower than control׳s bending modulus.

3 Significantly higher than the ISO minimum requirement of 50 MPa.

4 Significantly lower than control׳s bending strength.

**Table 2:** Results from compression testing. Results are reported as median ± 1 standard deviation.

**Compressive test**							
**Cement**	**Antibiotic**	**Amount of antibiotic added [g]**	**Mixing**	**Conditioning**	**Testing condition**	**Compressive modulus [MPa]**	**Compressive yield strength [MPa]**
Palacos	–	–	Vacuum	Ambient, saline, 21 days	Ambient	1559 ± 207.4	82.71 ± 63.52^2^
Palacos	Vancomycin	0.125	Vacuum	Ambient, saline, 21 days	Ambient	1543 ± 246.7	78.61 ± 47.91^2^^,^^3^
Palacos	Vancomycin	0.25	Vacuum	Ambient, saline, 21 days	Ambient	1279 ± 230.1^1^	77.01 ± 47.92^2^^,^^3^
Palacos	Vancomycin	0.50	Vacuum	Ambient, saline, 21 days	Ambient	1282 ± 301.2^1^	73.12 ± 33.34^2^^,^^3^
Palacos	Vancomycin	1.0	Vacuum	Ambient, saline, 21 days	Ambient	1098 ± 182.9^1^	69.62 ± 25.44^3^
Palacos	Vancomycin	2.0	Vacuum	Ambient, saline, 21 days	Ambient	980.7 ± 230.4^1^	61.72 ± 11.54^3^

1 Significantly lower than control׳s compressive modulus.

2 Significantly higher than the ISO minimum requirement of 70 MPa.

3 Significantly lower than control׳s compressive yield strength.

**Table 3:** Results from fracture toughness testing. Results are reported as median ± 1 standard deviation.

**Fracture toughness test**						
**Cement**	**Antibiotic**	**Amount of antibiotic added [g]**	**Mixing**	**Conditioning**	**Testing condition**	**Fracture toughness [MPa m**^**1/2**^**]**
Palacos	–	–	Vacuum	Ambient, saline, 21 days	Ambient	2.685 ± 0.091
Palacos	Vancomycin	0.125	Vacuum	Ambient, saline, 21 days	Ambient	2.627 ± 0.419
Palacos	Vancomycin	0.25	Vacuum	Ambient, saline, 21 days	Ambient	2.428 ± 0.267
Palacos	Vancomycin	0.50	Vacuum	Ambient, saline, 21 days	Ambient	2.467 ± 0.222
Palacos	Vancomycin	1.0	Vacuum	Ambient, saline, 21 days	Ambient	2.465 ± 0.166
Palacos	Vancomycin	2.0	Vacuum	Ambient, saline, 21 days	Ambient	2.187 ± 0.175

### 1.2 Drug elution data

See Table 4.

**Table 4:** Drug elution results over 60 days. Results (all units are mg) are reported as mean ± standard deviation (SD).

	**0.125 g**		**0.25 g**		**0.5 g**		**1.0 g**		**2.0 g**	
**Days**	**Mean**	**SD**	**Mean**	**SD**	**Mean**	**SD**	**Mean**	**SD**	**Mean**	**SD**
0	0.000	0.000	0.000	0.000	0.000	0.000	0.000	0.000	0.000	0.000
1	0.006	0.003	0.010	0.003	0.022	0.005	0.052	0.017	0.048	0.011
2	0.006	0.000	0.010	0.000	0.022	0.000	0.052	0.000	0.048	0.000
4	0.020	0.005	0.017	0.004	0.041	0.005	0.079	0.010	0.070	0.006
8	0.030	0.005	0.033	0.001	0.059	0.002	0.104	0.007	0.097	0.002
10	0.030	0.000	0.033	0.000	0.059	0.000	0.104	0.000	0.097	0.000
15	0.030	0.000	0.033	0.000	0.059	0.000	0.104	0.000	0.097	0.000
25	0.030	0.000	0.033	0.000	0.059	0.000	0.105	0.000	0.098	0.000
45	0.030	0.000	0.033	0.000	0.059	0.000	0.105	0.000	0.099	0.000
60	0.031	0.000	0.033	0.000	0.059	0.000	0.105	0.000	0.100	0.000

### 1.3 Antimicrobial activity testing

See Table 5, Table 6.

**Table 5:** Antimicrobial activity of eluted vancomycin (0.5 g) for three S. *aureus* strains. Results (all units are $\log_{10}\mathrm{CFU}/\mathrm{mL}$) (colony forming units, CFU) are reported as mean ± 1 standard deviation (SD).

	**ATCC 29213**		**n315**		**ATCC 33591**	
**Days**	**Mean**	**SD**	**Mean**	**SD**	**Mean**	**SD**
0	3.070	0.150	3.150	0.010	3.150	0.050
1	1.620	0.320	1.000	0.000	1.000	0.000
2	1.000	0.000	1.000	0.000	1.000	0.000
3	1.000	0.000	1.000	0.000	1.000	0.000
4	1.000	0.000	1.000	0.000	1.000	0.000
5	1.000	0.000	1.000	0.000	1.000	0.000
6	1.000	0.000	1.000	0.000	1.000	0.000
7	1.000	0.000	1.000	0.000	1.000	0.000
15	1.000	0.000	1.000	0.000	1.000	0.000

**Table 6:** Antimicrobial activity of eluted vancomycin (0.125–2.0 g) for S.epidermidis 35984. Results (all units are $\log_{10}\mathrm{CFU}/\mathrm{mL}$) are reported as mean ± 1 standard deviation (SD).

	**0.125 g**		**0.25 g**		**0.5 g**		**1.0 g**		**2.0 g**	
**Days**	**Mean**	**SD**	**Mean**	**SD**	**Mean**	**SD**	**Mean**	**SD**	**Mean**	**SD**
0	3.090	0.040	3.090	0.040	3.090	0.040	3.090	0.040	3.090	0.040
1	4.500	1.000	2.610	2.330	1.700	1.700	1.000	1.000	4.320	0.290
2	5.040	0.790	4.000	0.870	3.680	3.230	2.540	2.410	3.520	3.060
3	4.900	1.040	4.400	1.250	3.380	0.150	4.040	1.020	2.310	2.010
4	5.250	0.350	5.270	0.140	5.040	0.190	4.670	0.160	4.550	0.280
5	5.500	1.000	5.500	1.000	5.500	1.000	5.500	1.000	5.500	1.000
6	5.500	1.000	4.320	1.140	3.810	1.240	2.820	2.640	1.940	1.680
7	5.500	1.000	5.330	0.150	4.940	0.530	4.360	1.590	1.370	2.380
15	5.500	1.000	5.500	1.000	1.830	3.180	0.610	1.050	1.000	1.000

## 2 Experimental design, materials, and methods

### 2.1 Sample preparation

Cement, stored at 22 °C ± 1 °C, was prepared [2] with vacuum, − 50 to 100 mbar (Zimmer Compact Vacuum Cement Mixing System®). The six experimental groups were: control (Palacos® R Cement), and five treatments with 0.125 g, 0.25 g, 0.50 g, 1.0 g, and 2.0 g of vancomycin powder added to the polymer powder. Six samples per test were molded: drug elution disks (6 mm diameter × 4.5 mm height), compression cylinders (6 mm diameter × 12 mm height), four-point bend beams (75 mm × 10 mm × 3.3 mm), and fracture toughness beams (44 mm × 10 mm × 5 mm with crack length between 4.5 mm and 5.5 mm and width of 0.37 mm, Buehler® IsoMet^™^).

### 2.2 Mechanical testing

After 21 days in 1x PBS at 22 °C, mechanical testing was performed (Criterion C43.104, MTS Systems) according to ISO 5388 with force and displacement data recorded at 100 Hz. Displacement rate was 5 mm/min for four-point bending and compression tests and 10 mm/min for fracture toughness tests.

### 2.3 Release characteristic testing

Five cement disks per group, were submerged in 5 mL PBS in an incubator shaker at 37 °C and 100 rpm. At 1, 2, 4, 8, 10, 15, 25 and 45, and 60 days, 1.5 mL of the PBS was aspirated off, stored at − 20 °C, and samples were submerged in 5 mL fresh PBS. Vancomycin content was measured (high performance liquid chromatography with a C_18_ column [3]) in triplicate. Ten microliters of the sample was developed isocratically with 50 mM potassium phosphate buffer (pH 6.8) and acetonitrile (17:3) at a flow rate of 0.5 mL/min. Absorbance was monitored at 210 nm with peak intensity correlated to concentrations [4].

### 2.4 Antimicrobial activity testing

Three cement disks per group were sterilized (ethylene oxide gas), submerged in 3.4 mL tryptic soy broth (TSB; Becton Dickenson) and inoculated with bacteria (1000 CFU/mL): Methicillin-resistant Staphylococcus aureus (MRSA) n315 with a vancomycin minimum inhibitory concentration (MIC) of 0.5–1 mg/L [5], ATCC MRSA 33591 (vancomycin MIC 2 mg/L), ATCC *S. aureus* 29213 (vancomycin MIC 0.5 mg/L), and ATCC *S. epidermidis* 35984 (vancomycin MIC 1 mg/L) [6]. MRSA n315 was also tested at 10^6^ CFU/mL. TSB samples, taken at inoculation, daily for 7 days, and again at 14 days, were serially diluted (Mueller Hinton II agar plates, Sigma-Aldrich) for bacterial enumeration. Bacterial colonies were quantified after 18–24 hours incubation. All testing was performed in triplicate.
