# Supplementary material for: Data for vancomycin elution, activity and impact on mechanical properties when incorporated into orthopedic bone cement
Source: Data Brief. 2018 Jul 17;20:14–9. doi: 10.1016/j.dib.2018.07.028 (PMC6083008; doi:10.1016/j.dib.2018.07.028)
Supplement: Supplementary file 1 — Transparency document. [file mmc1.docx]

Conflicts of Interest: none
